# I2b2-etl: Python application for importing electronic health data into the informatics for integrating biology and the bedside platform

**DOI:** 10.1093/bioinformatics/btac595

**Published:** 2022-09-02

**Authors:** Kavishwar B Wagholikar, Layne Ainsworth, David Zelle, Kira Chaney, Michael Mendis, Jeffery Klann, Alexander J Blood, Angela Miller, Rupendra Chulyadyo, Michael Oates, William J Gordon, Samuel J Aronson, Benjamin M Scirica, Shawn N Murphy

**Affiliations:** Harvard Medical School, Boston, MA 02115, USA; Massachusetts General Hospital, Boston, MA 02114, USA; Mass General Brigham, Boston, MA 02199, USA; Brigham and Women’s Hospital, Boston, MA 02115, USA; Brigham and Women’s Hospital, Boston, MA 02115, USA; Mass General Brigham, Boston, MA 02199, USA; Harvard Medical School, Boston, MA 02115, USA; Massachusetts General Hospital, Boston, MA 02114, USA; Harvard Medical School, Boston, MA 02115, USA; Brigham and Women’s Hospital, Boston, MA 02115, USA; Mass General Brigham, Boston, MA 02199, USA; Mass General Brigham, Boston, MA 02199, USA; Mass General Brigham, Boston, MA 02199, USA; Harvard Medical School, Boston, MA 02115, USA; Mass General Brigham, Boston, MA 02199, USA; Brigham and Women’s Hospital, Boston, MA 02115, USA; Mass General Brigham, Boston, MA 02199, USA; Harvard Medical School, Boston, MA 02115, USA; Brigham and Women’s Hospital, Boston, MA 02115, USA; Harvard Medical School, Boston, MA 02115, USA; Massachusetts General Hospital, Boston, MA 02114, USA

## Abstract

**Motivation:**

The i2b2 platform is used at major academic health institutions and research consortia for querying for electronic health data. However, a major obstacle for wider utilization of the platform is the complexity of data loading that entails a steep curve of learning the platform’s complex data schemas. To address this problem, we have developed the i2b2-etl package that simplifies the data loading process, which will facilitate wider deployment and utilization of the platform.

**Results:**

We have implemented i2b2-etl as a Python application that imports ontology and patient data using simplified input file schemas and provides inbuilt record number de-identification and data validation. We describe a real-world deployment of i2b2-etl for a population-management initiative at MassGeneral Brigham.

**Availability and implementation:**

i2b2-etl is a free, open-source application implemented in Python available under the Mozilla 2 license. The application can be downloaded as compiled docker images. A live demo is available at https://i2b2clinical.org/demo-i2b2etl/ (username: demo, password: Etl@2021).

**Supplementary information:**

Supplementary data are available at *Bioinformatics* online.

## 1 Introduction

The i2b2 platform is deployed at major academic health institutions for querying for electronic health records (EHR) (Abend *et al.*, 2009; Murphy *et al.*, 2010). The platform provides a user-friendly interface that allows researchers with no expertise in information technology to find patient cohorts using EHR data. I2b2 has been deployed as a critical component of research networks including National Patient-Centered Clinical Research Network (PCORNet) (Klann *et al.*, 2018), Accrual for Clinical Trials (Visweswaran *et al.*, 2018) and Consortium for clinical Characterization of COVID-19 (4CE) (Brat *et al.*, 2020; Weber *et al.*, 2009).

The platform has been used for a wide spectrum of use cases including clinical-trial enrollment (Bucalo *et al.*, 2021), population management (Wagholikar *et al.*, 2019), biobanking (Castro *et al.*, 2021; Mate *et al.*, 2017; Segagni *et al.*, 2011), clinical decision support and epidemiological analysis (Klann and Murphy, 2013; Murchison *et al.*, 2021; Pfiffner *et al.*, 2016; Segagni *et al.*, 2011; Wagholikar *et al.*, 2017a, b). However, despite its impact and open-source availability, the deployment of the platform is largely limited to large academic medical centers.

A major obstacle for wider utilization of the i2b2 platform is the difficulty in data loading, as it requires the IT staff to learn the complex data-schema internal to the platform that has a star topology (Murphy *et al.*, 2007). To address this issue, we have developed the i2b2-etl package that specifies a simple input format and abstracts away the complex processes to initialize the internal i2b2 schema. This will facilitate wider deployment and utilization of the platform.

## 2 Materials and methods

We have implemented a Python application, referred to as ‘i2b2-etl’ that imports data in the Comma Separated Value (CSV) format into the i2b2 platform. The source code is available in open source (https://github.com/i2b2/i2b2-etl and https://github.com/i2b2/i2b2-etl-docker), and as compiled containers in Dockerhub. The application can be downloaded as a docker images, that are compatible with all common Linux distributions, Windows and Mac-OSX systems.

For an online demo see, https://i2b2clinical.org/demo-i2b2etl/ (username: demo, password: Etl@2021). After login navigates to ETL tab, press delete button and then choose upload files, selecting the CSV files from the Supplementary Material. Supplementary Appendix A provides the steps to install i2b2-etl. Supplementary Appendix B describes command-line interaction. Supplementary Appendix C demonstrates the use of Gitlab to use SQL queries as inputs to i2b2-etl.

**Fig. 1.**
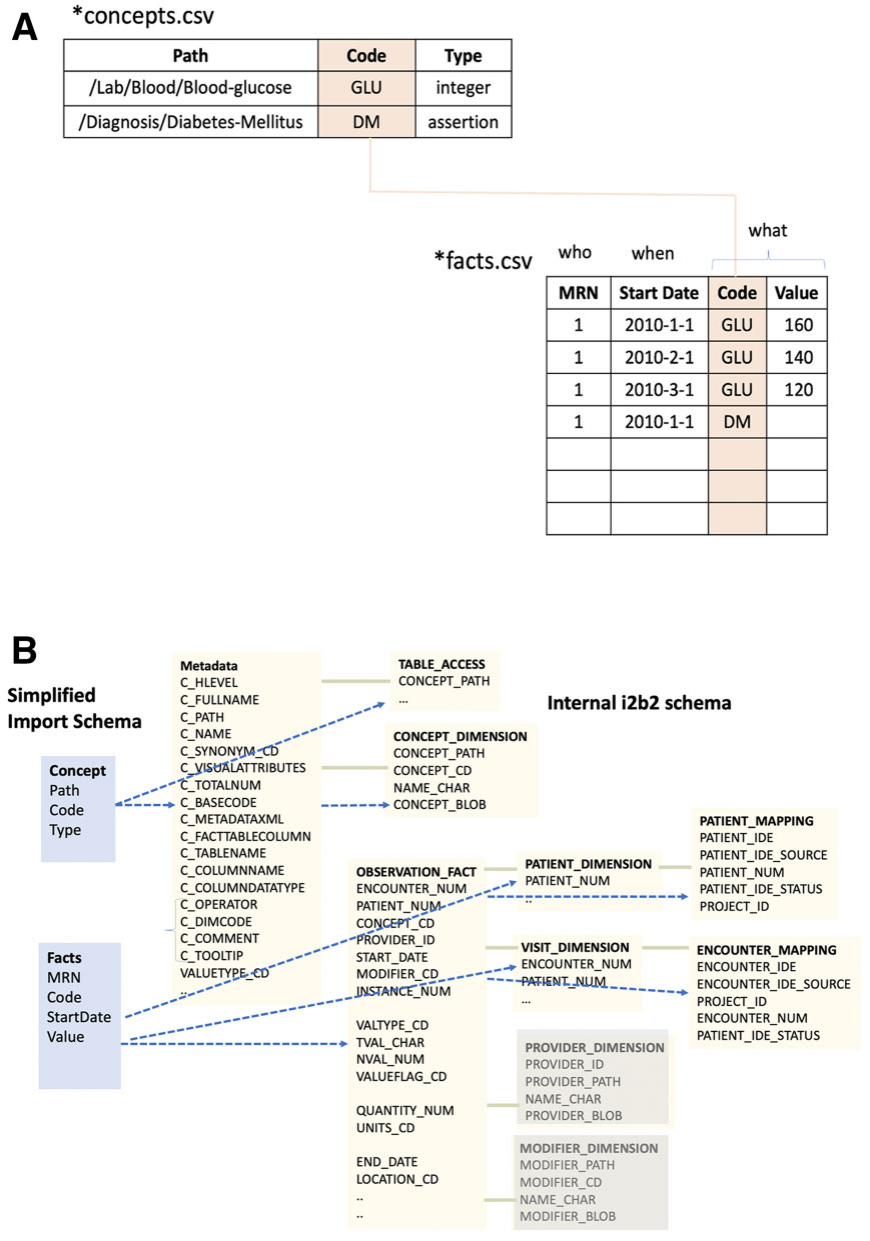
(**A**) I2b2-etl accepts two types of CSV files– concept files and fact files. Concept files provide the meta-data or dictionary for the creation of an ontology hierarchy. The fact files contain patient data. For example, the first row represents that patient with medical-record number 1, had a ‘GLU’ of 160 on 1st January 2010. The concepts file clarifies that GLU is blood-glucose, which is a laboratory test performed on the blood, as indicated in the path, and it has a value of an integer. I2b2-etl uses the concept files to validate the facts while performing the import. (**B**) I2b2-etl parses in the input schemas shown on the left (blue background) and executes processes to populate the internal i2b2 tables shown on the right (yellow background). The metadata, table access and concept dimension tables are essential for the functionality to display ontologies and to query patient data using ontologies. These are automatically populated by the i2b2-tool. The observation-fact and dimension tables are internal i2b2 tables in a star topology that contain EHR data, and the mapping tables serve to de-identify the patient record numbers. These tables (except the provider and modifier dimensions) are automatically generated by i2b2-etl. Without i2b2-etl, all these internal tables need to be populated individually, which requires an in-depth understanding of the schema, a challenge that is now resolved by i2b2-etl (A color version of this figure appears in the online version of this article.)

As shown in Figure 1A, i2b2-etl accepts two types of CSV files—concept files and fact files, which contain the meta-data and data, respectively.

Concept files provide the meta-data or dictionary for the creation of the ontology hierarchy. Concept files consist of three columns: path, code and type, and their names end in ‘_concepts.csv’. Each row in the concept file corresponds to a node in the ontology hierarchy displayed in the i2b2-User Interface. The path specifies the unique location of the node in the hierarchy. The type can be integer, float, assertion, string or large-string, and the code is the abbreviated reference to the concept. For example, in the first row in the concept file shown in Figure 1A, the path ‘/Lab/Blood/Blood-glucose’ leads to creation of the integer node called Blood-Glucose, as a child of the ‘Blood’ node. The ancestor nodes for ‘Lab’ and ‘Blood’ are automatically created.

The fact files contain patient data in four columns: medical record number (MRN), start-date, code and value. The name of fact files end in ‘_facts.csv’. Each row of the fact file provides the value of a specific observation (of a concept) for a patient referenced by the MRN starting at a particular point in time (start date). For example, the first row in the fact file shown in Figure 1A indicates that a value of 160 for blood glucose was observed on 1 January 2010, for the patient having a MRN 1.

I2b2-etl populates the tables in the internal i2b2 schema as elucidated in Figure 1B. The ontology hierarchy and SQL code snippets for ontology-based querying are auto-generated from the concept file and populated in the i2b2 metadata and concept dimension tables by the tool. The medical record numbers are converted into randomly generated integers and stored in the i2b2 patient-mapping table that is inaccessible to end-users. The latter can only access the randomized integers as patient numbers in the user interface. I2b2-etl performs validation of the input facts to ensure that each fact references a valid concept, has a value that conforms to its concept type, and that it has a valid time stamp.

We deployed i2b2-etl for a cardiovascular population health program at MassGeneral Brigham in Boston (Benson *et al.*, 2018; Blood *et al.*, 2020; Gordon *et al.*, 2021; Wagholikar *et al.*, 2019) The program involved daily interaction of navigators with patients. The resulting data were recorded in a relational database. To import this data into i2b2 for easy querying, we developed SQL queries to extract the project data into CSV files specified above. Next, we deployed i2b2-etl as a nightly job that executed the SQL queries and loaded the resulting CSV files into an i2b2 repository. The latter was setup specifically for the project using the i2b2 docker containers (Wagholikar *et al.*, 2018).

## 3 Results

Our i2b2-etl package allows importing of the ontology and patient-data as two simple CSV files. The cardiovascular program included data for 28 483 patients. Deployment of i2b2-etl resulted in 1395 concepts and over 4.7 million facts in the i2b2 repository, requiring 18 min for execution. The resultant i2b2-repository is used by the study staff to identify sub-cohorts in the population and to evaluate the program’s progress (Scirica *et al.*, 2021).

The novelty of i2b2-etl application is the simplified design of the input file schema, inbuilt de-identification and data validation. The input file schema abstracts away the complexity of the data schemas internal to the i2b2 platform (see Fig. 1B). This simplified mechanism will allow the IT staff at healthcare institutions to easily transform and load their institution’s EHR into the i2b2 platform.

Without i2b2-etl, the IT staff needs to thoroughly understand the complex schemas in i2b2 platform, in order to extract the data in conformance with the i2b2 schemas. As i2b2-etl can transform the simplified concept and file schemas into the platform’s schemas, IT staff is required to only focus on preparing SQL statements to yield the simplified input schemas. Moreover, with the integration of ETL module with Gitlab (Supplementary Appendix C), the entire ETL process can be automated, wherein Gitlab triggers the execution of SQL on the source database to extract the data as CSVs, which are then transformed into the i2b2 internal schemas and then loaded into the i2b2 platform’s database. Consequently, as the transform and loading steps are done by i2b2-etl, the IT staff only needs to focus on the extraction step, which can be performed by IT staff with minimal SQL expertise.

However, the simplified abstraction is at the expense of functionality in the i2b2 platform. I2b2-etl does not support querying using fields in the modifier, patient and visit dimensions of the i2b2 star topology. There are several alternative approaches that have been previously developed for importing EHR data into i2b2. Post *et al.* (2013a, b, 2016) have developed an application called Eureka that can load Excel files with custom schemas into the i2b2 database. Importing of EHR data into OMOP model has been used in several projects (Klann *et al.*, 2018, 2019; Majeed *et al.*, 2021; Rinner *et al.*, 2018) However, the major differentiator of i2b2-etl with the alternative approaches is the simplified abstraction for the input, which allows users without advanced training in data modeling to rapidly import EHR into the i2b2 platform, thereby improving accessibility of the EHR for secondary use cases.

## Funding

This work was supported by MassGeneral Brigham and National Institutes of Health [R00-LM011575, R01-HG009174 and R01HL151643].

*Conflict of Interest*: none declared.

## Supplementary Material

btac595_Supplementary_Data
