# Supplementary material for: I2b2-etl: Python application for importing electronic health data into the informatics for integrating biology and the bedside platform
Source: Bioinformatics. 2022 Sep 2;38(20):4833–6. doi: 10.1093/bioinformatics/btac595 (PMC9563689; doi:10.1093/bioinformatics/btac595)
Supplement: btac595_Supplementary_Data [file btac595_supplementary_data.zip › simple-etl-manuscript-supplementary-material.pdf]

Appendix A: Installation Instructions for ETL module

Prerequisites:

The install script requires docker-compose (see https://docs.docker.com/compose/install/)

Installation

git clone https://github.com/i2b2/i2b2-etl-docker

cd i2b2-etl-docker/postgres

docker-compose up -d i2b2-etl

verify that webclient is working

In web-browser open http://localhost/webclient

(username: demo , password: Etl@2021)

After login navigate to ETL tab. Here press delete button, and then choose upload files, selecting the csv files from the sample-data folders in the source code (https://github.com/i2b2/i2b2-etl/tree/master/sample-data)

Video tutorial: https://i2b2.github.io/i2b2-etl-docker/etl-tutorial

Appendix B: Command Line Instructions

i2b2-etl provides a command line interface and API interface to import, delete concepts and facts and encounters.

Deploy i2b2-etl as docker container on i2b2-installation

For installing i2b2 and the ETL container see see https://github.com/i2b2/i2b2-etl-docker

$ docker run -it –-name i2b2-etl -v /tmp:/tmp i2b2/i2b2-etl:1.0.1

Executing the I2B2-ETL

Start a bash shell inside the i2b2-etl container

$ docker exec -it i2b2-etl bash

For ease of documentation use etl as an alias for command invocation

$ alias etl="python -m i2b2_cdi ${ARGS}"

I2B2-ETL commands

Help

This will list all possible operation of i2b2-etl

$ etl --help

OR

$ etl -h

Delete concepts

$ etl concept delete -c <env-file>

Load concepts

$ etl concept load -c <env-file> -i <input-dir>

Note: File name should have pattern like *_concepts.csv

Delete facts

$ etl fact delete -c <env-file>

Load facts

Load facts with concept_cd validation.

$ etl fact load -c <env-file> -i <input-dir>

Load facts with no validation.

$ etl fact load -c <env-file> -i <input-dir> --disable-fact-validation

Note: File name should have pattern like *_facts.csv

Delete patients

$ etl patient delete -c <env-file>

Load patients

$ etl patient load -c <env-file> -i <input-dir>

Create project and user

Create new project & user along with password , also assign new project to new user.

$ etl project add -c <config-file> --project-name <project-name> --project-user-password <project-user-password>

Load data into project

Copy data from one project to another.

$ etl project load -c <config-file> --project-name <project-name>

Change user password

Change password for user in i2b2

$ etl project password -c <config-file> --user <user-id> --password <password>

Appendix C: Gitlab integration

Automate ETL by integrating Gitlab-containers with i2b2

Installation

1. git clone https://github.com/i2b2/i2b2-etl-docker
2. cd i2b2-cdi/postgres/
3. sudo docker-compose up -d i2b2-etl gitlab-runner i2b2-mssql

Wait for 2-5 minutes for containers to start

4. verify that webclient is working on http://localhost/webclient (username: demo, pass: Etl@2021)
5. Verify that Gitlab is available on http://localhost:8090/ (username: root and password: adminadmin)

Video tutorial

https://i2b2.github.io/i2b2-etl-docker/gitlab-tutorial
